# Side-By-Side Evaluation of Three Commercial ELISAs for the Quantification of SARS-CoV-2 IgG Antibodies

**DOI:** 10.3390/v14030577

**Published:** 2022-03-11

**Authors:** Philipp Girl, Sonja Mantel, Heiner von Buttlar, Roman Wölfel, Katharina Müller

**Affiliations:** 1 Bundeswehr Institute of Microbiology, Neuherbergstraße 11, 80937 Munich, Germany; sonjamantel@bundeswehr.org (S.M.); heinervonbuttlar@bundeswehr.org (H.v.B.); romanwoelfel@bundeswehr.org (R.W.); katharina5mueller@bundeswehr.org (K.M.); 2 German Centre for Infection Research (DZIF), Partner Site Munich, 80937 Munich, Germany

**Keywords:** SARS-CoV-2, COVID-19, quantification, antibodies

## Abstract

In December 2020, WHO presented the first international standard (WHO IS) for anti-SARS-CoV-2 immunoglobulin. This standard is intended to serve as a reference reagent against which serological tests can be calibrated, thus creating better comparability of results between different tests, laboratories, etc. Here, we have examined three different commercial ELISA kits for the quantification of SARS-CoV-2 IgG antibodies, namely the Anti-SARS-CoV-2 QuantiVac ELISA (IgG) (Euroimmun, Lübeck, Germany), the SERION ELISA agile (Institut Virion Serion, Würzburg, Germany), and the COVID-19 quantitative IgG ELISA (DeMediTec Diagnostics, Kiel, Germany). According to the manufacturers, all are calibrated against the WHO IS and can provide results in either international units (IU) (DeMediTec) or arbitrary antibody units (BAU) per milliliter (Euroimmun, Virion Serion), which are numerically identical, according to the WHO. A total of 50 serum samples from vaccinated individuals were tested side by side and according to the manufacturer’s instructions. We compared the test results of all three assays with each other to assess comparability and with a quantitative in-house virus neutralization test (micro-NT). In summary, our data are consistent with other studies published on this topic that tested similar assays from different manufacturers. Overall, the agreement between quantitative ELISAs is variable and cannot be used interchangeably despite calibration against a standard. Therefore, interpretation of results must still be individualized and tailored to each case. More importantly, our results highlight that quantitative ELISAs in their current form cannot replace neutralization tests.

## 1. Introduction

In December 2020, the WHO established and provided the first International Standard (WHO IS) for anti-SARS-CoV-2 immunoglobulin. This standard is intended to serve as a reference reagent against which secondary standards, such as those used, for example, in commercial serological assays, can be calibrated [1]. The calibration can then facilitate a better comparison of test results between different assays, laboratories, etc. Such comparability of serological test results is of particular importance in order to be able to define limit values for immune protection in the future. This is also one of the bases for further recommendations for (booster) vaccinations [2].

## 2. Materials and Methods

Here, we investigated three different commercial ELISA kits for the quantification of SARS-CoV-2 IgG antibodies, i.e., Anti-SARS-CoV-2-QuantiVac-ELISA (IgG) (Euroimmun, Lübeck, Germany), SERION ELISA agile (Institut Virion Serion, Würzburg, Germany) and COVID-19 quantitative IgG ELISA (DeMediTec Diagnostics, Kiel, Germany). According to the manufacturers, all are calibrated against the WHO IS and can give results in either international units (IU) (DeMediTec) or arbitrary binding antibody units (BAU) per milliliter (Euroimmun, Virion Serion), which according to the WHO are numerically identical [3]. Table 1 shows the main characteristics of all three assays as provided by the manufacturers.

A total of 50 serum samples (duplicates) from vaccinated individuals (taken three weeks after the second vaccine dose) were manually tested side by side and according to the manufacturer’s instructions using 10-fold serial dilutions (starting at 1:101). We compared test results of all three tests with each other to evaluate comparability as well as with a quantitative in-house virus neutralization assay (micro-NT). Micro-NT was performed as previously described using the early SARS-CoV-2 strain MUC-IMB-1 (clade B1) [4]. All samples were residual diagnostic material. Therefore, no further information (e.g., demographic characteristics, vaccine characteristics etc.) could be assigned to individual samples.

## 3. Results

### 3.1. Comparability of Test Results

**Figure 1:**
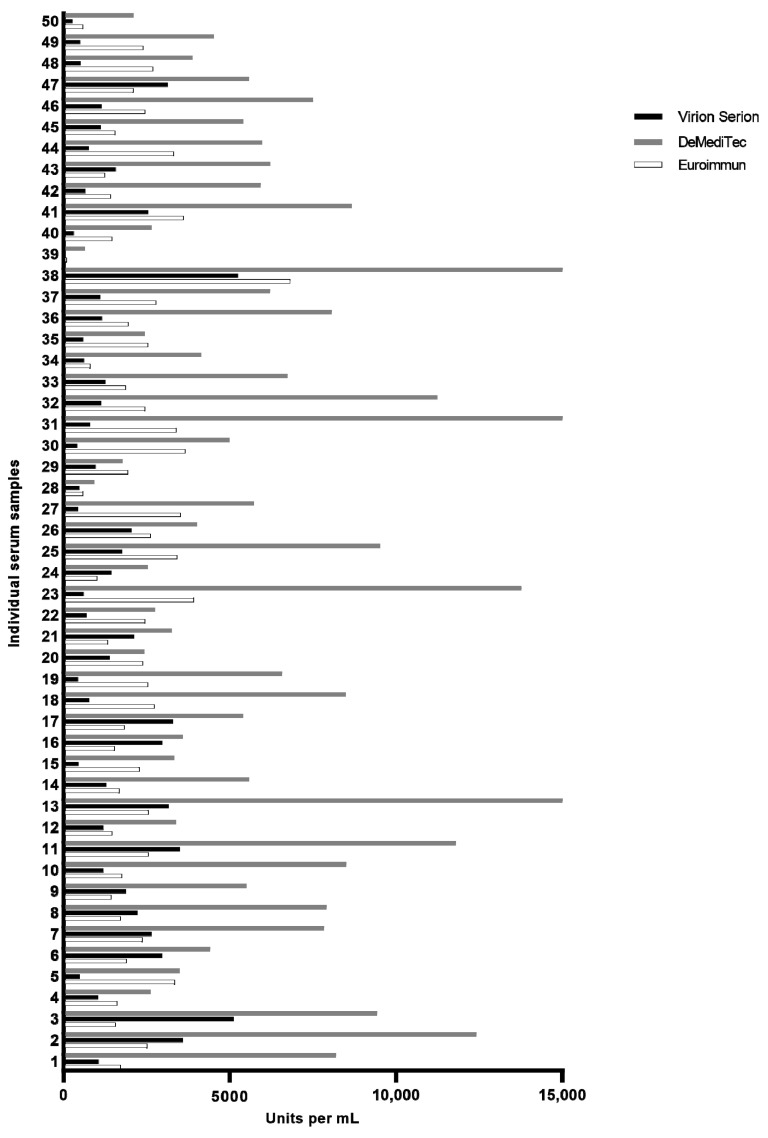
Quantification of anti-SARS-CoV-2 antibodies by all three tests. X-axis gives units per mL, by definition BAU and IU are numerically identical.

Quantitative evaluation results are shown comparatively in Figure 1. We found significant differences (*p* < 0.0001) in the amounts of antibodies detected by each test: The DeMediTec assay almost consistently yielded the highest test results with antibody amounts of up to 16,755 IU/mL (median 5587 IU/mL; 95% CI 5223–7423), whereas the other two detected considerably lower amounts in almost all samples with a maximum of 5250 BAU/mL (Virion Serion; median 1140 IU/mL; 95% CI 1173–1868) and 6810.2 BAU/mL (Euroimmun; median 2198 IU/mL; 95% CI 1927–2541), respectively.

All three tests consistently detected the highest and lowest amounts of antibodies in the identical sample set. Notably, the sample with the lowest antibody level (i.e., sample 39) was also the sample with the lowest NT titer. Interestingly, while the DeMediTec test and the Euroimmun test both detected low antibody levels in this sample, it was measured as negative in the Virion Serion test. However, the agreement of the individual measurements of the remaining 48 samples between the three tests was moderate at best. The highest correlation was observed between the Euroimmun and the DeMediTec tests (Spearman *r* = 0.51, *p* < 0.0002). A similar correlation was also observed between the DeMediTec and the Virion Serion (Spearman *r* = 0.45, *p* = 0.0009) whereas no significant correlation could be observed between the Euroimmun and the Virion Serion tests (Spearman *r* = 0.01, *p* = 0.9).

### 3.2. Correlation between Quantitative Values and Neutralizing Antibody Titers

**Figure 2:**
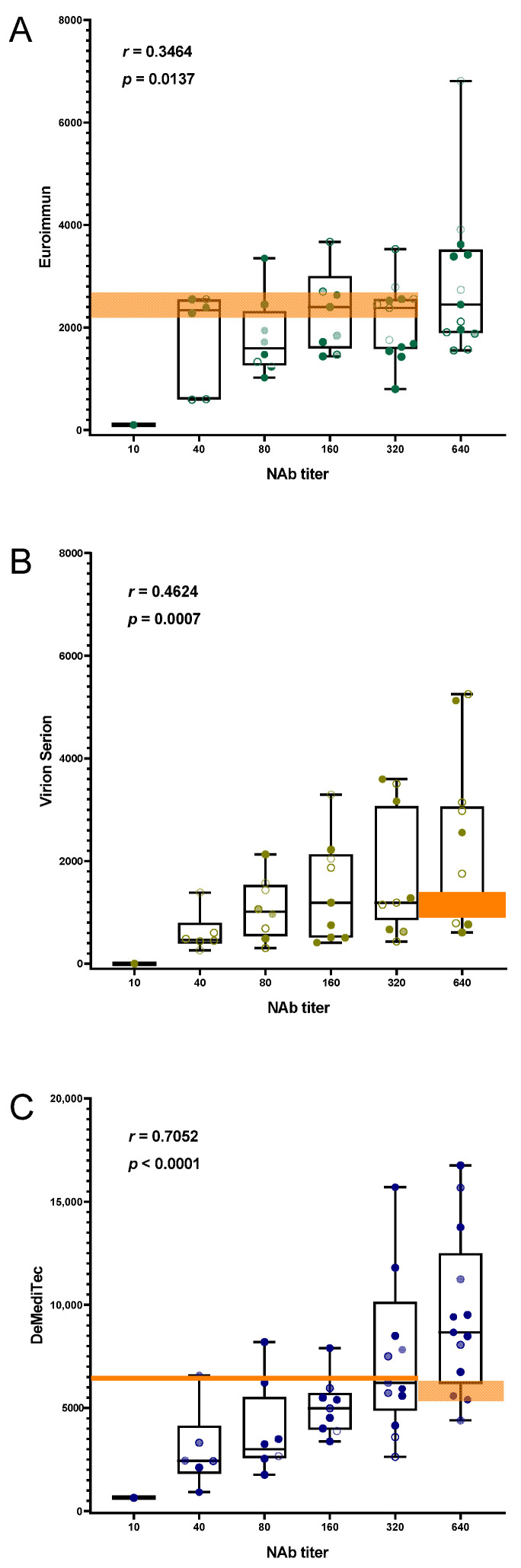
Distribution of immunoassay results and neutralizing antibody titers. Spearman’s rank coefficients (*r*) and their corresponding *p* values are indicated for each assay. Whiskers extend from minimal to maximal values and all points are shown. Agreement between Euroimmun and NT (**A**) as well as Virion Serion and NT (**B**) was fair to middling while it was good between DeMediTec and NT (**C**). The highlighted areas indicate exemplary samples with significantly different NT titers despite apparently very similar ELISA antibody unit readings.

We also compared test results of each immunoassay with the results obtained with a quantitative micro-NT whereby titers ranged between 10 and 640 (median titer: 296). Direct comparison of NT titer and assay results generally indicated a positive and significant correlation for all three tests with NT (Figure 2). Nonetheless, Spearman rank coefficients differed notably and tended to be lower for both Euroimmun/NT (*r* = 0.35, *p* = 0.01) and Virion Serion/NT (*r* = 0.46, *p* = 0.0007) (Figure 2A,B), whereas it was the highest for DeMediTec/NT (*r* = 0.71, *p* < 0.0001) (Figure 2C).

## 4. Discussion

The WHO IS is a pool of plasma samples from eleven convalescent patients intended to facilitate a better comparison of test results between different assays. It was assigned a dimensional unit of 1000 International Units per milliliter (IU/mL) for neutralization activity as well as a numerically identical arbitrary Binding Antibody Unit (BAU/mL) specific for each viral antigen (e.g., the Spike (S) protein, the S1/S2 domain or the nucleoprotein (N)) [1]. Interestingly, when the standard became available manufacturers did not directly adopt this standardization. Instead, they started providing an experimental conversion factor to convert the tests original units into WHO units (i.e., BAU, IU). In this study, we performed a head-to-head comparison of three commercial quantitative SARS-CoV-2 ELISAs from different manufacturers, all calibrated against the WHO IS. In addition, we also evaluated the relationship between assay results and a quantitative in-house micro-NT.

Overall, the described antigen specificity of the BAU could make it difficult to achieve comparability between assays targeting different antigens. Nonetheless, a collaborative study published in 2020 reported a strong harmonization of ELISA assays by using the WHO IS despite differences in target antigens [5]. Concerning the tests used in this study, all three detect IgG antibodies and use the S protein, or at least parts of it, as antigen. While the S1 domain of the S protein is the only antigen used by Euroimmun, the Virion Serion uses whole S protein as antigen. Interestingly, the Virion Serion has no significant agreement with the S1 specific Euroimmun, which could be explained by the differences in antigen. This is also supported by the fact that we observed better agreement between the Virion Serion and the DeMediTec, which also uses the whole S protein as antigen. However, it should be noted that this correlation was only moderate, which could be due to the fact that the DeMediTec uses not only whole but also trimeric S protein.

At the same time, we observed a lower correlation between the DeMediTec and the Euroimmun. Overall, more data are required and until then, quantitative ELISA results should be continued to be interpreted as antigen specific and should only be compared carefully with each other.

Of all three tests, the DeMediTec produced the highest results almost across the board. Interestingly, this is also the only test that reports results in IU and not BAU. The indication in IU is unusual, since the unit IU is basically intended by the WHO for neutralization tests and not for binding tests. However, according to WHO, both units are numerically identical. Results should therefore be directly comparable despite the different units. However, we could not confirm this on the basis of our results. In theory, a good correlation between the amounts of antibodies and NT titer could help avoid labor-intensive and time-consuming neutralization assays and thus improve patient management. Previous studies reported a good correlation between IgG antibodies determined by ELISA and neutralizing activity [6,7]. We also observed fairly good correlation (*r* = 0.71) between the antibody amounts detected by the DeMediTec and the NT. However, agreement between the Virion Serion and the NT was notably lower (*r* = 0.46) and even worse between the Euroimmun and the NT (*r* = 0.34). This observation could also be due to the different antigens used, which is supported by the fact that the best correlating DeMediTec uses trimeric S protein (which most closely corresponds to viral S protein) while the least correlating Euroimmun uses only the S1 domain.

It is important to note that even an overall good correlation does not automatically mean that this test can replace the NT. On the contrary, when looking at the individual results in detail, it becomes clear that no conclusion can be drawn from the amount of antibodies measured to the NT titer and vice versa. This is exemplified in Figure 2 in the highlighted areas, which illustrate that samples with comparable antibody levels nevertheless show large differences in NT titer. The same is also shown by the large span of whiskers. Interestingly, this large span was independent of whether overall agreement with NT was better or worse.

## 5. Conclusions

In summary, our data are in line with other studies published on this topic testing the same or similar assays from various manufacturers [7,8,9,10,11]. Overall, agreements between quantitative ELISAs vary and while they could be useful to assess patients’ antibody responses to SARS-CoV-2, they cannot be used interchangeably despite the calibration against a standard. At the same time, this also highlights that interpretation of results must continue to be assay-specific and personalized. Even more importantly, our results underline that quantitative ELISAs in their current state cannot replace neutralization assays. In addition, our results emphasize the need for further unification of the standard as well as cooperation between manufacturers to improve the cross-utility of their tests as other groups have previously suggested.

## Figures and Tables

**Table 1:** General assays information provided by the manufacturers.

*MANUFACTURER*	*KIT ASSAY*	*TARGET ANTIGEN*	*METHOD*	*IMMUNOGLOBLIN DETECTED*	*UNITS*	*ALTERNATIVE WHO UNITS*	*CONVERSION FACTOR*	*CUT-OFF*	*DYNAMIC RANGE*
Euroimmun	Anti-SARS-CoV-2 QuantiVac ELISA (IgG)	S1 ^1^	ELISA	IgG	RU/mL	BAU/mL	3.2	32	19.2–256
Virion Serion	SERION ELISA agile SARS-CoV-2 IgG	S ^2^	ELISA	IgG	U/mL	BAU/mL	2.1	31.5	10.5–525
DeMediTec	COVID-19 (SARS-CoV-2) quantitative IgG ELISA	S ^3^	ELISA	IgG	AU/mL	IU/mL	4.5	49.5	43–690

RU = relative units, AU = arbitrary units, BAU = binding antibody units, IU = international units; ^1^ recombinantly produced in human cells, based on SARS-CoV-2 isolateWuhan-Hu-1, ^2^ whole S protein, ^3^ whole S protein, trimeric.

## Data Availability

The data presented in this study are available on request from the corresponding author. The data are not publicly available due to due to privacy regulations.
